# Four-six year clinical follow-up of deep brain stimulation for obsessive compulsive disorder

**DOI:** 10.1192/bjo.2021.195

**Published:** 2021-06-18

**Authors:** Himanshu Tyagi

**Affiliations:** UCL Institute of Neurology, Queen Square, The National Hospital for Neurology and Neurosurgery

## Abstract

**Background:**

Over the past 20 years a number of robust studies have established the clinical effectiveness and safety of Deep brain stimulation (DBS) in adults with profound multi-treatment-refractory obsessive-compulsive disorder (OCD). However long term (>12 months) outcomes with this novel neurosurgical intervention are still inadequately reported. Our group conducted the first UK study of DBS in OCD between 2013-2017. All participants in our trial achieved a responder status at 15 month endpoint and the main results were reported in 2019. A specialist multidisciplinary clinic was established after the trial to provide life-long aftercare in the form of scheduled clinical and hardware reviews. Here we are reporting a preliminary analysis of the long-term clinical, functional and social outcomes from this cohort.

**Method:**

Long term follow-up clinical data (15–75 months, 2015 onwards) were prospectively collected from the participants who were enrolled in the original MRC-UCL pilot study of DBS for OCD. DBS parameters, battery health and status, social circumstances, mental state and medication adjustments were noted alongside the outcome measures of YBOCS at clinical follow-up encounters. Additional ratings of GAF, SDS and certain qualitative measures were recorded at least once every year since initial study completion.

**Result:**

Five out of six participants continued with DBS treatment and kept responder status. One participant had his DBS switched off and hardware removed. One participant had multiple hospital admissions to manage comorbidity progression to primary condition. One participant had OCD severity scores revised upwards despite continuing gains in QoL. Secondary outcomes generally matched the 15 month end point of initial trial. All participants experienced minor to major changes in their relationships with partners or family. Qualitative feedback indicated that DBS was well tolerated by 5/6 subjects but the burden of specialist follow-up remained significant.

**Conclusion:**

Our long term follow-up data indicate that DBS is safe and conferred a sustained long-term benefit in reduction of obsessive-compulsive symptoms. A non-trivial burden of checking and maintenance of implanted hardware, comorbidity-unmasking following successful OCD treatment, perceived ‘burden of normality’ by the participant, need for life-long follow-ups with specialist multidisciplinary team including DBS nurses, highly specialist psychiatrists from National OCD service, neuropsychiatrists, neurologists and neurosurgeons partially counterbalances the gains offered by this treatment. Overall DBS offers a safe, effective and enduring alternative to participants who do not respond to any other form of OCD treatment and do not wish to undergo ablation surgery.

